# Dual Roles of Quercetin in Platelets: Phosphoinositide-3-Kinase and MAP Kinases Inhibition, and cAMP-Dependent Vasodilator-Stimulated Phosphoprotein Stimulation

**DOI:** 10.1155/2012/485262

**Published:** 2012-12-17

**Authors:** Won Jun Oh, Mehari Endale, Seung-Chun Park, Jae Youl Cho, Man Hee Rhee

**Affiliations:** ^1^Laboratory of Physiology & Cell Signaling, College of Veterinary Medicine, Kyungpook National University, Daegu 702-701, Republic of Korea; ^2^Department of Veterinary Pharmacology and Toxicology, College of Veterinary Medicine, Kyungpook National University, Daegu 702-701, Republic of Korea; ^3^Department of Genetic Engineering, Sungkyunkwan University, Suwon 440-746, Republic of Korea

## Abstract

*Background*. Progressive diseases including cancer, metabolic, and cardiovascular disorders are marked by platelet activation and chronic inflammation. Studies suggest that dietary flavonoids such as quercetin possess antioxidant, anti-inflammatory, and antiplatelet properties, which could prevent various chronic diseases including atherosclerosis and thrombosis. However, the mechanism and the signaling pathway that links quercetin's antiplatelet activity with its anti-inflammatory property is limited and thus further exploration is required. The aim of this paper was to examine the link between antiplatelet and anti-inflammatory roles of quercetin in agonist-induced platelet activation. *Methods*. Quercetin effects on agonist-activated platelet-aggregation, granule-secretion, [Ca^2+^]_i_, and glycoprotein-IIb/IIIa activation were examined. Its effects on PI3K/Akt, VASP, and MAPK phosphorylations were also studied on collaged-activated platelets. *Results*. Quercetin dose dependently suppressed collagen, thrombin, or ADP-induced platelet aggregation. It significantly inhibited collagen-induced ATP release, P-selectin expression, [Ca^2+^]_i_ mobilization, integrin-*α*_IIb_ *β*_3_ activation, and augmented cAMP and VASP levels. Moreover, quercetin attenuated PI3K, Akt, ERK2, JNK1, and p38 MAPK activations, which were supported by platelet-aggregation inhibition with the respective kinase inhibitors. *Conclusion*. Quercetin-mediated antiplatelet activity involves PI3K/Akt inactivation, cAMP elevation, and VASP stimulation that, in turn, suppresses MAPK phosphorylations. This result suggests quercetin may have a potential to treat cardiovascular diseases involving aberrant platelet activation and inflammation.

## 1. Introduction

Platelets play a major role in hemostasis and thrombosis [1], in which the latter causes a serious problem leading to myocardial infarction, atherosclerosis, ischemia, and stroke [2]. At the sites of vascular damage, platelet activation by agonists such as collagen, adenosine diphosphate (ADP), and thrombin resulted in an increase in [Ca^2+^]_i_ concentration, platelet shape change, secretion, and aggregation [3]. Activated platelets also release various aggregation mediators including ADP, adenosine triphosphate (ATP), thromboxane A2 (TXA2), serotonin, and various proteins [4]. These released mediators stimulate G-protein coupled receptors (GPCRs) that are necessary for phospholipase C (PLC), protein kinase C (PKC), phosphoinositide-3 kinase (PI3K) [4], and MAP kinases activations [5]. Furthermore, activated platelets secret P-selectin, which stabilizes the initial *α*_IIb_ *β*_3_ integrin-fibrinogen binding to more stable platelet aggregate formation [6] and links platelet activation with inflammation [7].

Platelet activation and chronic inflammation are sequels of a wide range of progressive diseases, including cancer, metabolic and cardiovascular disorders [8], suggesting that prevention of inflammation and aberrant platelet activation by dietary flavonoids, such as quercetin, is one of the ways to prevent various chronic diseases including atherosclerosis and thrombosis [8–11]. Several animal and clinical studies have suggested that flavonoids such as quercetin are rich in fruits, vegetables, red wine, and tea where consuming them may protect the development of cardiovascular disease risks through their antioxidant and anti-inflammatory properties [10–14]. The results of population studies [11, 12], and animal and clinical intervention studies [10, 13, 14] using quercetin-rich diets, have suggested antiatherosclerosis and antithrombosis effects of quercetin through their antioxidant, antiplatelet, and anti-inflammatory properties. Studies have reported an inverse association between dietary quercetin intake and mortality from coronary heart disease [10, 11]. Quercetin may also be a promising dual antiplatelet and anti-inflammatory/antiatherosclerosis agent that warrants comprehensive evaluation of its potential as a new lead class of drug development. However, its mechanism of action and signaling pathways that links quercetin's antiplatelet property with its anti-inflammatory activity is limited in platelets and further exploration is required to add data on the existing reports and expand the knowledge base about the relationship of the antiplatelet and anti-inflammatory properties of the compound in association with cardiovascular disorders.

Previous reports indicated that quercetin possesses a potent antioxidant, immunomodulatory, anti-inflammatory, and antiatherosclerotic and antiplatelet properties [15, 16]. It has also been reported to inhibit MAPKs, Akt, Src, JAK-1, and Tyk2 activations [17]. However, the modulatory effects of quercetin in agonist-induced platelet activation, protein and/or lipid kinases phosphorylations, and cyclic nucleotide activities are only partially explored. In addition, information on the effect of quercetin in aberrant platelet activation and inflammation is limited. In this study, therefore, we determined that quercetin inhibits agonist-induced platelet activation through inhibition of PI3K/Akt activation with subsequent cAMP elevation and VASP stimulation that, in turn, suppresses ERK2, JNK1, and p38 MAPK phosphorylations.

## 2. Materials and Methods

### 2.1. Materials

Collagen and ADP were purchased from Chronolog (Havertown, PA, USA). Thrombin, Fura-2/AM, quercetin and forskolin and 3-isobutyl-1-methyl xanthine (IBMX), SB203580, SP600125, and PD98059, LY-294002, and wortmannin were procured from Sigma (St. Louis, MO, USA). ATP assay kit was obtained from Biomedical Research Service Center University (Buffalo, NY, USA). Mouse monoclonal to CD62P antibody and goat polyclonal to mouse IgG antibody (FITC) were obtained from Abcam (Cambridge, UK). Antibodies to phospho-p44/42, total-p44/42, phospho-p38, total-p38, phospho-JNK, total JNK, phospho-Akt, total Akt, phospho-PI3K p85/p55, PI3 kinase p85, phospho-VASP (Ser 157), and *β*-actin were purchased from Cell Signaling (Beverly, MA, USA). Monoclonal antibody to VASP (phosphorylated) (pSer239) (16C2) was obtained from Enzo Life Sciences (PA, USA). Alexa Fluor 488 fibrinogen conjugate was obtained from Molecular Probes (Eugene, OR, USA). Cyclic AMP Kit was procured from Ann Arbor (MI, USA). All other chemicals were of reagent grade.

### 2.2. Platelet Preparation

Blood was collected from the abdominal artery of 8~10 weeks old rats with citrate phosphate dextrose solution (CPD; 90 mM Na_3_C_6_H_5_O_7_·2H_2_O, 14 mM C_6_H_8_O_7_·H_2_O, 128.7 mM NaH_2_PO_4_·H_2_O, 2.55 g/100 mL dextrose). Platelet-rich plasma (PRP) was prepared by centrifugation of the blood samples at 1000 rpm for 7 min twice, and platelets were washed with washing buffer. Washed platelets were then gently resuspended in Tyrode buffer (137 mM NaCl, 12 mM NaHCO_3_, 5.5 mM glucose, 2 mM KCl, 1 mM MgCl_2_, 0.3 mM NaHPO_4_, pH 7.4) to a final concentration of 5 × 10^8^ platelets/mL.

### 2.3. Platelet Aggregation

Washed 5 × 10^8^ platelets/mL were preincubated for 3 min at 37°C in the presence of 1 mM exogenous CaCl_2_ with or without various concentrations (12.5–100 *μ*M) of quercetin, and then platelet aggregation was stimulated by collagen (2.5 *μ*g/mL), ADP (10 *μ*M), or thrombin (0.1 U/mL). The aggregation was monitored by using an aggregometer (Chronolog, Havertown, PA, USA) at a constant stirring of 1200 rpm and aggregation rates were measured as the light transmission changes were recorded for 8 min.

### 2.4. P-Selectin Secretion

P-selectin (CD62) expression on platelets was measured using FITC-labeled anti-CD62P antibody. Quercetin-pretreated platelets were activated by collagen and incubated for 5 min at 37°C with stirring condition. Washed platelets were then centrifuged followed by resuspension in ice-cold PBS containing 10% FBS, and 1% sodium azide. Samples were blocked with ice-cold PBS containing 3% BSA and labeled with CD62P primary antibody for 30 min at 4°C in dark condition. The sample was washed repeatedly in ice-cold PBS and labeled with FITC-conjugated secondary antibody in 3% BSA/PBS for 30 min at 4°C in the dark. After repeated washing with ice-cold PBS, the sample was resuspended in ice-cold PBS, 3% BSA, and 1% sodium azide. Flow cytometry was performed using FACSCalibur flow cytometer (Becton Dickinson, San Jose, CA, USA), and data was analyzed using CellQuest software (Becton Dickinson Immunocytometry System, San Jose, CA, USA).

### 2.5. Measurement of ATP Release

ATP secretion from the dense granules of platelet was determined in a luminometer (GloMax 20/20, Promega, Madison, WI, USA) using the ATP assay kit (Biomedical Research Service Center, Buffalo, NY, USA) according to the manufacturer's protocol. Briefly, platelets were incubated for 3 min at 37°C with or without various concentrations of quercetin and then stimulated with collagen for 5 min. The reaction was stopped, platelets were centrifuged, and supernatants were used for the assay. 

### 2.6. Determination of Cytosolic-Free Ca^2+^ Concentration

Platelets were prepared as described above and incubated with 5 *μ*M fura-2/AM at 37°C for 60 min. Fura 2-loaded platelets (5 × 10^8^ platelets/mL) were preincubated for 1 min at 37°C with various concentrations of quercetin in the presence of 1 mM CaCl_2_ and then stimulated with thrombin for 200 seconds. Fura-2 fluorescence was measured with a spectrofluorimeter (F-2500, Hitachi, Japan) in an excitation wavelength altering every 0.5 sec from 340 nm to 380 nm; the emission wavelength was at 510 nm. Then, the [Ca^2+^]_i_ was estimated using the method of Blaustein [18].

### 2.7. Determination of Fibrinogen Binding

Washed platelets were initially treated with quercetin or vehicle and incubated for 5 min at room temperature. Two hundred *μ*g/mL Alexa Fluor 488-human fibrinogen were added before collagen (2.5 *μ*g/mL) stimulation and then the sample was incubated at 37°C for 15 min. Alexa Fluor 488-fibrinogen binding to platelets was determined by flow cytometry using FACScan flow cytometer (Becton Dickinson, San Jose, CA, USA), and data were analyzed using CellQuest software (Becton Dickinson Immunocytometry Systems, San Jose, CA, USA). Fibrinogen nonspecific binding was estimated by measuring its binding in the presence of a specific integrin inhibitor, RGDS peptide (1 mM). 

### 2.8. Immunoblotting

Platelets (5 × 10^8^/mL) were activated with collagen for 5 min in the presence of 1 mM CaCl_2_ with or without quercetin (12.5, 25, 50, and 100 *μ*M) and immediately dissolved in sample buffer (0.125 M Tris-HCL at pH 6.8, 2% FBS, 2%  *β*-mercaptoethanol, 20% glycerol, 0.02% bromophenol blue in the presence of 1 mM phenylmethylsulfonylfluoride (PMSF), 2 *μ*g/mL aprotinin, 1 *μ*g/mL leupeptin, and 1 *μ*g/mL pepstatin). Protein concentration was determined using BCA assay (PRO MEASURE, iNtRON biotechnology, Korea) on ice. After boiling for 5 min, the proteins were resolved by electrophoresis in 10% SDS-PAGE and then transferred to PVDF membranes in a transfer buffer (25 mM Tris (pH 8.5) and 20% methanol). Membrane was blocked with 5% skim milk, washed, and subjected to immunoblotting with antiphospho ERK1/2, anti-ERK1/2, antiphospho p38, anti-p38, antiphospho JNK, anti-JNK, antiphospho AKT, anti-AKT, antiphospho-p55, antiphospho-p85 PI3K, anti-PI3K, antiphospho-VASP^Ser157^, and antiphospho VASP^Ser239^ antibodies. The immunoblots were again incubated with HRP secondary antibody and the membranes were visualized using enhanced chemiluminescence, ECL (iNtRON Biotechnology, Korea).

### 2.9. Measurement of cAMP

Platelets were preincubated at 37°C for 1 min and treated with quercetin (25, 50, and 100 *μ*M) or FSK (1 *μ*M) and incubated for 5 min at stirring condition. The mixture was boiled for 5 min and cooled at 4°C. Then, the precipitate was centrifuged and supernatant used to determine the cyclic AMP content using EIA kits (Ann Arbor, MI, USA) following acetylation as described by the manufacturer.

### 2.10. Statistical Analysis

Data were analyzed by one-way ANOVA, using Statistical Analysis Software, version 9.1 (SAS Institute Inc., Cary, NC, USA) tool, followed by a *post hoc* Dunnett's test in order to determine the statistical significance of the differences between treatment groups. All data are presented as means ± SEM, and *P* ≤ 0.05 were considered to be statistically significant.

## 3. Results

### 3.1. Quercetin Inhibits Agonist-Induced Platelet Aggregation

**Figure 1:**
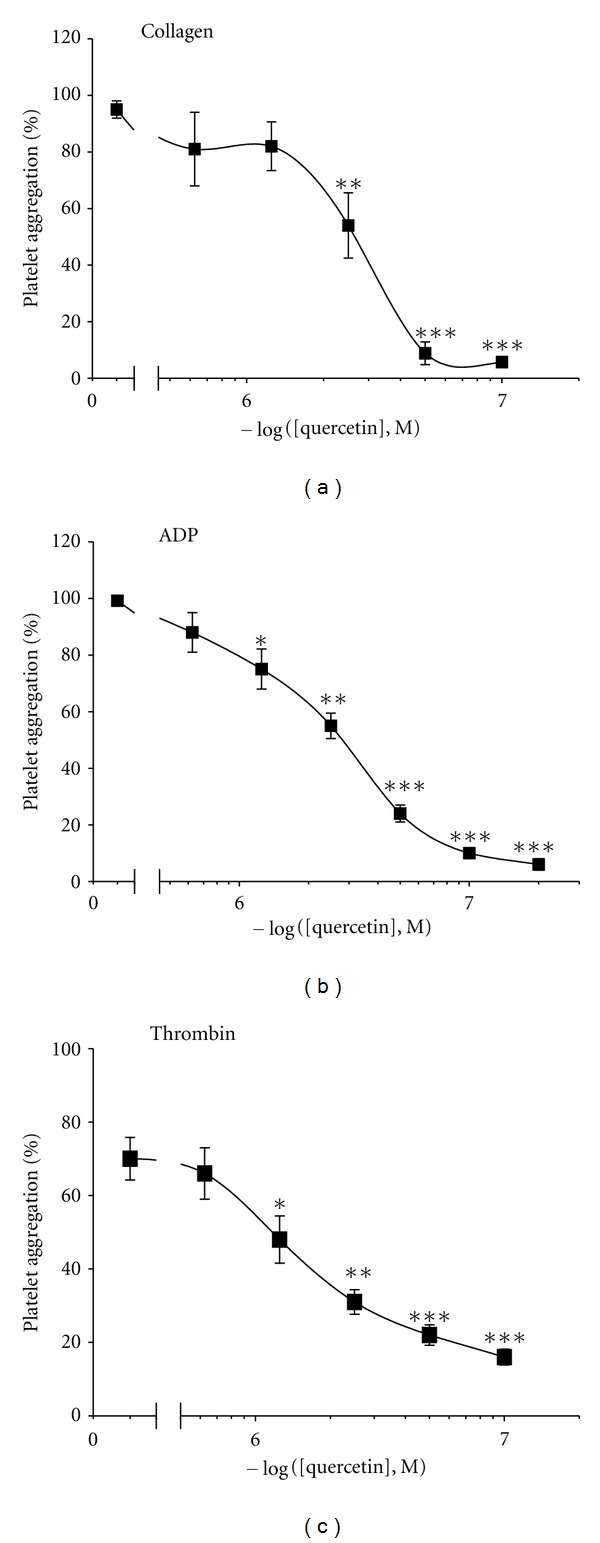
Quercetin inhibits agonist-induced platelet aggregation. Platelets were preincubated with quercetin in the presence of 1 mM CaCl_2_ for 2 min at 37°C and stimulated with collagen (a), ADP (b), and thrombin (c). Aggregation was terminated at 5 min and percent aggregation was determined. Tracings ((a), (b), and (c)) are summary of 8 to 10 experiments with mean ± SEM of at least 8 independent experiments. *P < 0.05, **P < 0.01 or ***P < 0.001 versus agonist activated control.

Quercetin inhibited platelet aggregation induced by collagen (2.5 *μ*g/mL), ADP (10 *μ*M), and thrombin (0.1 U/mL), respectively (Figure 1). The fifty percent inhibitory concentrations (IC_50_) of quercetin to the above indicated agonists-activated platelet aggregations were estimated to be 25.0 ± 4.4, 25.0 ± 3.1, and 12.5 ± 3.1 *μ*M (Figures 1(a), 1(b), and 1(c)), respectively.

### 3.2. Quercetin Reduces Agonist-Induced ATP Release, P-Selectin Expression, and [Ca^2+^]_i_ Mobilization

**Figure 2:**
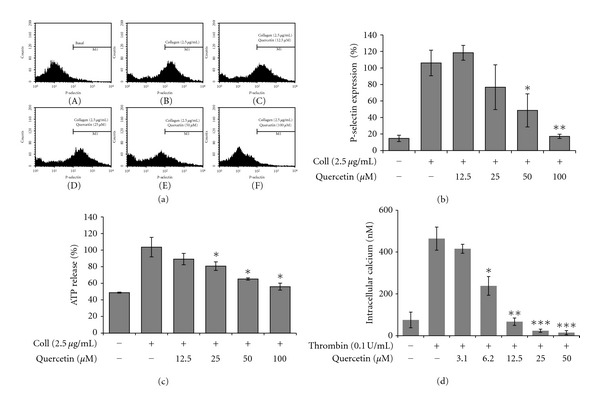
Quercetin influences collagen-activated granule secretions. Platelets were preincubated with quercetin and stirred in an aggregometer for 2 min before collagen or thrombin stimulation for 5 min and the reaction was terminated followed by granule secretion assay. ((a) and (b)) Effect of quercetin on collagen-induced P-selectin expression. (a) Panels ((A) and (B)) represent untreated and collagen stimulated, and ((C)–(F)) represent quercetin dose-dependent effects. (b) The bar graph shows summary of 4 independent experiments. (c) ATP release in response to agonist stimulation was performed as described in the “Materials and Methods.” (d) Platelets (3 × 10^8^/mL) were loaded with Fura-2/AM and preincubated with or without quercetin in the presence of 1 mM CaCl_2_ for 2 min followed by thrombin (0.1 U/mL) stimulation for 5 min at 37°C and [Ca^2+^]_i_ levels were determined. Bar graphs show mean ± SEM of at least 4 independent experiments performed. *P < 0.05, **P < 0.01 or ***P < 0.001 versus agonist-activated control.

Since granule secretions are crucial early events of platelet activation, we examined the influence of quercetin treatment on collagen-induced dense and *α*-granule secretions. As shown in Figure 2, quercetin reduced collagen-induced P-selectin secretion (Figures 2(a) and 2(b)) and ATP release (Figure 2(c)) in a dose-dependent manner, respectively. In addition, it significantly attenuated thrombin evoked [Ca^2+^]_i_ mobilization in the concentrations indicated in Figure 2(d).

### 3.3. Quercetin Increases Platelet cAMP Levels and Enhances Vasodilator-Stimulated-Phosphoprotein (VASP) Phosphorylation

Cyclic AMP generation and cyclic nucleotide-dependent protein kinase activity are known to be inhibited by platelet activation [3], and agents that can enhance cAMP reverse platelet activation. We, therefore, investigated whether quercetin influences platelet cAMP levels. Quercetin markedly increased the level of cAMP in collagen-stimulated platelets (Figure 4(a)). Besides, we further assessed effect of quercetin with adenylyl cyclase activator and phosphodiesterase inhibitor on platelet aggregation. As such, coincubation of low-dose quercetin with forskolin (2.5 *μ*M), adenylyl cyclase activator or IBMX (50 *μ*M), broad spectrum cyclic phosphodiesterase inhibitor, highly potentiated quercetin-mediated platelet aggregation inhibition and augmented individual effects upon combination (Figures 4(c) and 4(d)). 

Since VASP, a substrate of cyclic nucleotide- (cAMP/cGMP-) dependent protein kinases (PKA/PKG), inhibits agonist-induced platelet aggregation [19], we examined the effect of quercetin in platelet VASP expression. Though no basal VASP expression was detected (Figure 4(b)), quercetin treatment dose dependently increased VASP^Ser157^ and VASP^Ser239^ phosphorylations with increased translocation of VASP^157^ from 46 to 50 kDa protein. This suggests that quercetin has a role in stimulating cyclic nucleotide-dependent protein kinase mediated VASP phosphorylation. 

### 3.4. Quercetin Reduces Fibrinogen Binding to Activated Integrin *α*_IIb_ *β*_3_

**Figure 3:**
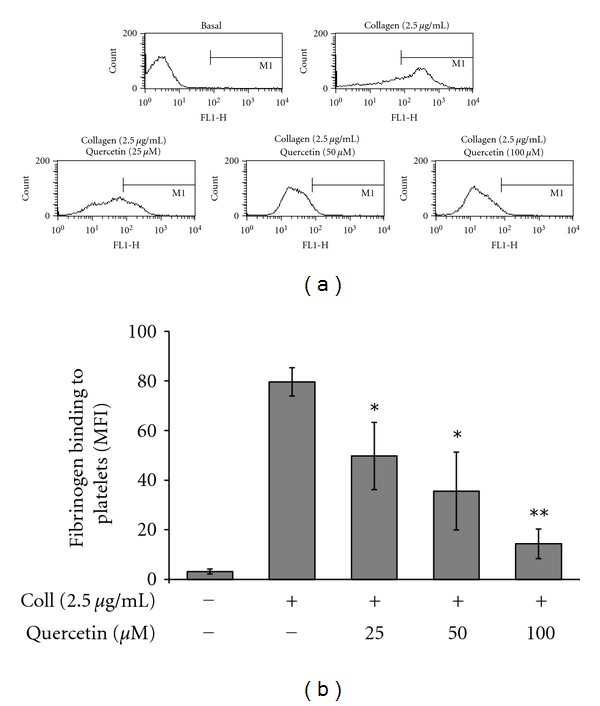
Quercetin attenuates fibrinogen binding to integrin *α*_IIb_ *β*_3_. Fibrinogen binding to integrin *α*_IIb_ *β*_3_ in platelets pre-treated with quercetin and stimulated by collagen (2.5 *μ*g/mL) together with Alexa Fluor 488-human fibrinogen (200 *μ*g mL^−1^) followed by incubation at 37°C for 15 min. (a) Tracings are representatives of 4 independent experiments. (b) Bar graph represents summary of quercetin effects on fibrinogen binding. *P < 0.05, **P < 0.01.

**Figure 4:**
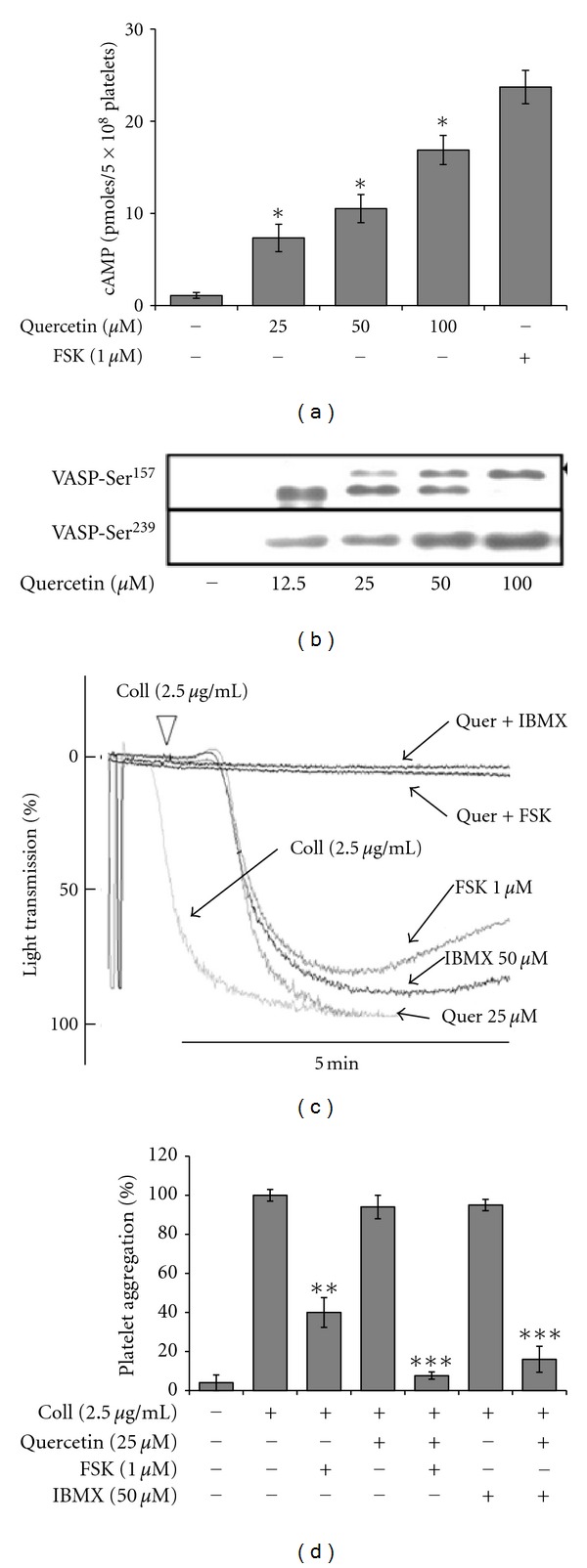
Quercetin enhances basal cyclic AMP levels and VASP phosphorylation. Rat platelets were stirred with either the presence of vehicle or quercetin in an aggregometer, the reaction was terminated, and cAMP enzyme immunoassays and western blot for VASP activation were performed. (a) Dose-dependent quercetin effects in resting platelets cAMP levels. (b) Dose-dependent effect of quercetin on VASP activation. ((c) and (d)) Forskolin and IBMX before treatment strongly potentiated quercetin-induced platelet aggregation. Results are summary of at least 3 independent experiments performed and bar graphs presented as mean ± S.E.M. *P < 0.05, **P < 0.01 or ***P < 0.001 versus control.

The ligand-binding functional change of integrin *α*_IIb_ *β*_3_ is the main outcome of adhesion and activation in platelets [20] followed by aggregation as a result of the adhesive substrates bound to the membranes of activated platelets [21]. Thus, we examined the role of quercetin on functional response of integrin *α*_IIb_ *β*_3_ activation. Collagen-induced fibrinogen binding to its receptor was dose dependently reduced in quercetin-treated platelets (Figures 3(a) and 3(b)). This finding suggests that quercetin may impair integrin *α*_IIb_ *β*_3_ conformational changes for high affinity fibrinogen binding site exposure (inside-out signaling) that occurs as a result of prior platelet agonist interactions. 

### 3.5. Quercetin Suppresses Collagen-Stimulated Platelet MAP Kinase Phosphorylations

**Figure 5:**
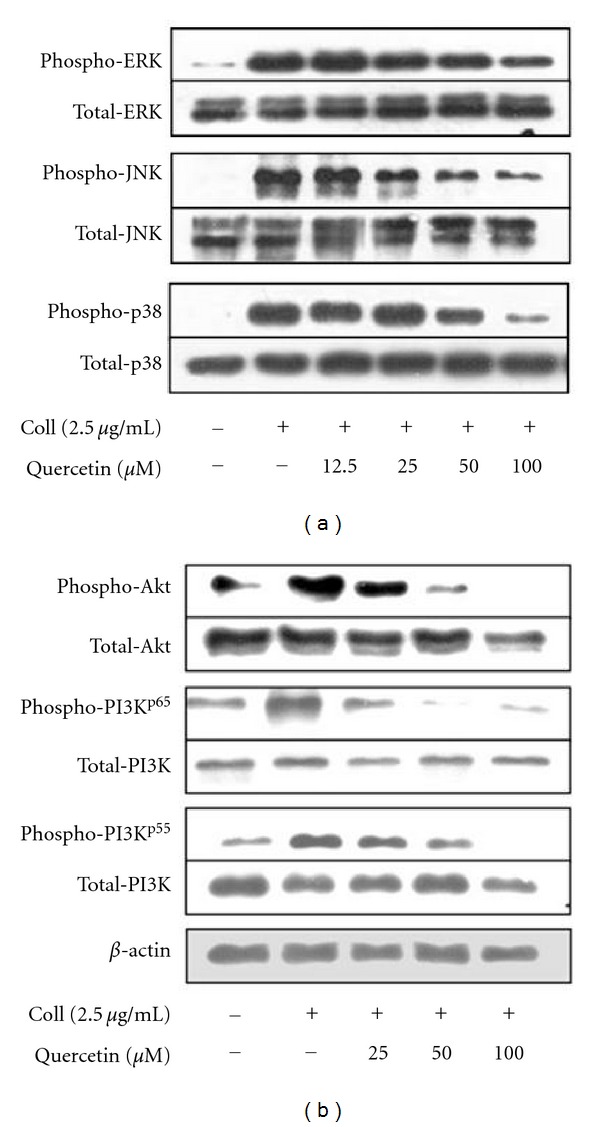
Quercetin suppresses collagen-activated PI3K/Akt and MAP kinase phosphorylations. ((a) and (b)) Washed platelets were stirred in an aggregometer with quercetin or vehicle at the concentrations indicated for 3 min and stimulation with collagen for 5 min before termination of the reactions. Proteins were extracted, separated by SDS-PAGE transferred to PVDF membranes, and immunoblotted with antibodies. Blots were visualized by ECL. (a) Quercetin dose-dependent effect on p38, JNK1, and ERK2 phosphorylations. (b) Effects of quercetin on platelet PI3K and Akt phosphorylation. Immunoblots are representatives of 3 to 4 experiments.

Quercetin is known to inhibit MAPKs, and the presence of p38 MAPK, ERK, and JNK has been demonstrated in blood platelets and reported to be phosphorylated by various platelet agonists [22]. As a result, we thought to determine whether collagen-induced MAPK phosphorylations are affected by quercetin. Our findings show that quercetin markedly inhibited collagen-stimulated ERK, JNK, and p38 MAP kinases in a dose-dependent manner (Figure 5(a)). The involvement of the above indicated MAP kinases in the antiplatelet activity of quercetin was further confirmed by using the respective inhibitors (PD98059 (30 *μ*M), SB203580, and SP600125 (10 *μ*M)) in collagen-induced platelet aggregation, respectively (data not shown).

### 3.6. Quercetin Arrests PI3K/Akt Signaling

Quercetin is a known inhibitor of PI3K and is a parent compound from which LY294002 (PI3K inhibitor) was derived, and PI3K plays a crucial role in platelet function such as activation, adhesion, spreading, and aggregation [23], with Akt, the main target of PI3K signaling [4]. Thus, the effect of quercetin on collagen-induced platelet PI3K/Akt activation was examined. Interestingly, quercetin significantly and dose dependently suppressed collagen-induced platelet Akt and PI3K phosphorylations (Figure 5(b)). Further, wortmannin or LY294002 (PI3K inhibitors, 20 *μ*M) suppressed platelet adhesion and activation via reducing [Ca^2+^]_i_ mobilization and *α*_IIb_ *β*_3_ activation (data not shown).

## 4. Discussion

Quercetin is known to be a negative regulator of cardiovascular disease risks as consumption of this compound is related to reduced incidences of stroke [24] and myocardial infarction [25]. Apart from its antioxidant activity [26], a multitude of anticipated mechanisms for quercetin mediated reduction of such risks have been reported. These mechanisms include inhibition of platelet activation [27], thrombus formation [28, 29], 5-HT secretion, and TXA2 release or binding to its receptor [30, 31]. In addition, inhibition of tyrosine [27], lipid [32], and serine/threonine kinases [33] have been reported as quercetin effects. In this regard, the ability of quercetin to bind competitively with ATP at the nucleotide binding site makes the compound an inhibitor of several protein kinases [32]. As a result, it was used as a lead compound to develop LY294002 and other inhibitors of PI3K [34]. A critical consideration for quercetin-mediated inhibition of platelet function may lie on the link between its anti-inflammatory property and antiplatelet activity. However, data on the relationship of quercetin-mediated antiplatelet effects with cAMP and/or PDE activity as well as MAPK and PI3K/Akt phosphorylation is scarce. 

We in the present study showed that quercetin suppressed the main pathways involved in platelet aggregation through inhibition of agonist-induced platelet activation, [Ca^2+^]_i_ mobilization, granule secretion, and fibrinogen binding. Our results also showed that quercetin inhibited collagen-induced PI3K and Akt phosphorylations downstream of collagen receptor. This effect was supported by suppressive effect of PI3K-inhibitors (wortmannin or LY294002) to platelet activation via inhibition of [Ca^2+^]_i_ mobilization and *α*_IIb_ *β*_3_ activation. An enhanced integrin *α*_IIb_ *β*_3_ receptor binding to fibrinogen is particularly considered to be the final common pathway for platelet aggregation [35]. In accordance to the present study, quercetin is reported to inhibit collagen-induced PI3K, Akt, and PLC*γ* activations and [Ca^2+^]_i_ mobilization in platelets [27, 36]. Akt is the key downstream molecule of PI3K signal that can be phosphorylated by collagen-induced platelet activation [37] and thrombus formation [23] where its inhibition by quercetin may have a negative role in platelet function. 

In the present study, quercetin significantly elevated cAMP-mediated VASP phosphorylation in resting platelets and addition of IBMX increased this effect further. Such an effect may provide a sound rationale for considering quercetin as a potential antiplatelet therapy in combination with cAMP elevating agents or alone. An increase in intracellular cAMP concentration either through enhancing adenylyl cyclase (AC) or suppressing phosphodiesterase (PDE) has been reported to inhibit platelet responses activated by various agonists such as collagen, thrombin, ADP, and TXA2 [38] or attenuate the [Ca^2+^]_i_ mobilization, which is an essential factor for platelet aggregation [3]. VASP phosphorylation has also been reported to inhibit integrin *α*_IIb_ *β*_3_ activation and platelet aggregation [39]. The proposed mechanism of quercetin action in this study may include inhibition of PI3K/Akt pathway with a subsequent increase in cAMP-mediated VASP phosphorylation, and a reduction in [Ca^2+^] mobilization. Recent reports indicated that Akt activation decreased cAMP levels through increment of PDE activity [40, 41]. On the other hand, cAMP-elevating agents such as cilostamide and cilostazol (PDE3 inhibitors) or forskolin (AC activator) are reported to show inhibitory effects to the PI3K-Akt signaling pathway in collagen-stimulated platelets [42]. This study, however, did not rule out whether PI3K/Akt or cyclic nucleotide pathway is upstream signaling and if the latter involves negative feedback mechanism. Thus, exploring the exact mechanism of interaction between the two signaling pathways in the presence of quercetin requires further investigation. 

Our findings in this report show that quercetin attenuated p38, JNK1, and ERK2 phosphorylations in collagen-activated platelets. The involvement of ERK2 p38 and JNK1 signalings on the antiplatelet activity of quercetin was further confirmed by using the respective MAPK inhibitors in collagen-induced platelet aggregation. This result suggests that the antiplatelet effect of quercetin may be linked to its anti-inflammatory effect as its pretreatment involves inhibition of MAPK activation in collagen-induced platelets. We have thus established in this paper that the inhibitory effect of quercetin on platelet activation by collagen might be through inhibition of PI3K/Akt stimulation, induction of cAMP-mediated VASP phosphorylation, and inhibition of MAPKs activation. This is in line with a previous study indicating an inhibition of adenylyl cyclase-mediated MAP kinase phosphorylation in collagen-stimulated platelets [43]. In addition, PDE-inhibitor induced reduction of platelet aggregation and integrin *α*_IIb_ *β*_3_ activation is reported to be mediated by inhibition of MAPK and Akt activation [44]. Interestingly, quercetin-mediated attenuation of P-selectin expression and MAP kinase activation in this study suggests that the antiplatelet activity of the compound could be linked to its regulation of hemostatic- and inflammatory responses. Since platelets are involved in inflammation, P-selectin expression on the membrane of activated platelets is the main link between platelets and inflammatory cells [7, 45] and quercetin-mediated suppression of P-selectin expression and MAPKs activation in this paper may be attributed to its anti-inflammatory property. 

Extensive studies, using various experimental setups, have indicated the role of ERK2, JNK1, and p38 in platelet granule secretion and aggregation [22, 46]. Using collagen [47] and thrombin [48] as agonists, previous reports indicated the involvement of ERK2 activation in platelet secretion and aggregation as well as JNK1 phosphorylation in thrombus formation [49]. Besides, P38 activation has been shown in collagen- [50] or thrombin-induced [51] platelet activation and secretion, which was restored by p38 inhibitors [52]. Platelet aggregation and thrombus formation are also known to involve in MAP kinase activation [53] and platelet-platelet cross-linking of fibrinogen bound to activated-*α*_IIb_ *β*_3_ [54]. Thus, the dual antiplatelet and anti-inflammatory properties of quercetin in the present study may have a role in treating aberrant platelet activation as an antiatherothrombotic and anti-inflammatory agent. Therefore, The inhibitory property of quercetin on agonist-induced granule secretion, [Ca^2+^]_i_ mobilization, *α*_IIb_ *β*_3_, PI3K/Akt and MAP kinases activations, and an enhanced cAMP-dependent VASP phosphorylation in platelet aggregation reflects the potential use of the compound as a candidate dual antiplatelet, anti-inflammatory agent. 

In conclusion, this study suggests that the inhibitory property of quercetin in platelet aggregation may involve (i) inhibition of PI3K/Akt signaling, (ii) induction of cAMP-mediated VASP phosphorylation, and (iii) inhibition of the ERK2, p38, and JNK1 MAP kinase phosphorylations in activated platelets. Thus, the ability of quercetin to inhibit [Ca^2+^]_i_ mobilization, integrin activation, ATP release, and P-selectin expression during platelet aggregation, in combination with its anti-inflammatory effects, suggests that quercetin could be considered as an antiatherothrombosis and anti-inflammatory agent. Given the observed effects of quercetin on platelet signaling and functional responses, it will be important to identify the specific active metabolite that is responsible for the observed effects that link PI3K/Akt and MAPK inhibition and cAMP-dependent VASP activation. This will enable a more detailed mode of action at the molecular level to be determined, and the therapeutic potential of quercetin supplementation to be assessed.
